# Our Sincere Thanks

**Published:** 1881-02

**Authors:** 


Our Sincere Thanks are Due in an especial manner to those gentlemen to whom the first number of the current volume was sent, inviting them to become subscribers to the Journal, for their prompt and kind compliance with our request; and we beg to assure them of our determination to render the succeeding issues worthy of their patronage and encouragement. That the issue would reach some who would not be capable of appreciating the *motive* for sending it nor the value of the publication, was confidently anticipated ere it was mailed to their address. But the number of those who have authorized the enrollment of their names on our subscribers' list and sent us words of cheer, so greatly out-number and counter-balance the few who decline, that we feel highly complimented and encouraged in our efforts for the new volume.

